# The Elongin Complex Antagonizes the Chromatin Factor Corto for Vein *versus* Intervein Cell Identity in Drosophila Wings

**DOI:** 10.1371/journal.pone.0077592

**Published:** 2013-10-21

**Authors:** Julien Rougeot, Myrtille Renard, Neel B. Randsholt, Frédérique Peronnet, Emmanuèle Mouchel-Vielh

**Affiliations:** 1 Université Pierre et Marie Curie-Paris 6, UMR7622, Paris, France; 2 Centre National de la Recherche Scientifique, UMR7622, Laboratoire de Biologie du Développement, Paris, France; University of Bern, Switzerland

## Abstract

*Drosophila* wings mainly consist of two cell types, vein and intervein cells. Acquisition of either fate depends on specific expression of genes that are controlled by several signaling pathways. The nuclear mechanisms that translate signaling into regulation of gene expression are not completely understood, but they involve chromatin factors from the Trithorax (TrxG) and Enhancers of Trithorax and Polycomb (ETP) families. One of these is the ETP Corto that participates in intervein fate through interaction with the *Drosophila* EGF Receptor – MAP kinase ERK pathway. Precise mechanisms and molecular targets of Corto in this process are not known. We show here that Corto interacts with the Elongin transcription elongation complex. This complex, that consists of three subunits (Elongin A, B, C), increases RNA polymerase II elongation rate *in vitro* by suppressing transient pausing. Analysis of phenotypes induced by *EloA*, *B*, or *C* deregulation as well as genetic interactions suggest that the Elongin complex might participate in vein *vs* intervein specification, and antagonizes *corto* as well as several TrxG genes in this process. Chromatin immunoprecipitation experiments indicate that Elongin C and Corto bind the vein-promoting gene *rhomboid* in wing imaginal discs. We propose that Corto and the Elongin complex participate together in vein *vs* intervein fate, possibly through tissue-specific transcriptional regulation of *rhomboid*.

## Introduction

*Drosophila* wings are mainly composed of two cell types, vein and intervein cells. In *Drosophila melanogaster*, vein cells form a stereotyped network of five longitudinal veins and two cross-veins that act as rigid supports necessary for flight. Intervein cells are much more abundant than vein cells and separate veins from each other. They are less pigmented and die shortly after adult emergence, whereas vein cells survive into adulthood. Intervein cells from the two apposed wing monolayers strongly adhere *via* integrins. By contrast, vein cells do not adhere to each other, and thus form fluid-conducting tubes surrounded by intervein tissue [1].

Intervein and vein cells acquire their identities during wing imaginal disc development from the third larval through the pupal stage. This process relies on several signaling pathways, including the *Drosophila* EGF receptor (DER) – MAP kinase ERK pathway (for a review, see [2]). While initial wing disc proliferation is induced by global activation of DER-ERK signaling, specification and differentiation of vein and intervein cells require fine-tuning of this pathway. During the third larval instar, longitudinal veins are first determined as broad regions called proveins, each expressing a specific combination of transcription factors that provides positional information. A subset of these factors induces localized expression of *rhomboid* (*rho*) in provein cells, which directs them to acquire vein fate [3]–[5]. Rho is part of a positive feedback loop on the DER-ERK pathway: Rho-mediated proteolytic processing of DER ligands leads to ERK activation in future vein cells [6]-[8] and higher DER-ERK signaling in turn increases *rho* expression [9]. Whereas *rho* is required for development of vein cells, *blistered* (*bs*), which encodes a homolog of the mammalian Serum Response Factor (SRF), is expressed in future intervein cells and controls acquisition of intervein cell fate [10], [11]. *bs* is repressed by DER-ERK signaling in provein territories, and represses in turn *rho* in future intervein cells during the pupal stage [12]. Acquisition of vein or intervein cell identity thus depends on the outcome of a fine-tuned balance between *rho* and *bs* expression, that both are regulated by the DER-ERK pathway.

Activation of the DER-ERK pathway induces diphosphorylation of ERK that can then enter the nucleus, but little is known about the nuclear mechanisms that link activated, phosphorylated ERK to regulation of gene expression. Interestingly, several studies in different organisms have connected MAP kinase signaling to chromatin factors from either the Trithorax Group (TrxG) or the Enhancers of Trithorax and Polycomb (ETP) family [13]-[16]. TrxG proteins form multimeric complexes that bind chromatin, deposit specific post-translational histone modifications, interact with the transcriptional machinery, and/or remodel nucleosomes. TrxG complexes maintain an open chromatin conformation and counteract transcriptional repression mediated by Polycomb complexes (PcG). Hence, TrxG complexes mostly maintain active gene expression (for reviews, see [17], [18]). However, TrxG complexes BAP and PBAP, *Drosophila* counterparts of the yeast chromatin remodeling complex SWI/SNF, can also participate in transcriptional repression [19]–[22]. ETPs are PcG and TrxG co-factors involved in both PcG silencing and TrxG activation, and might thus participate in a switch between activation and repression of transcription (for a review, see [23]). Several TrxG complexes are involved in control of vein *vs* intervein fate. Indeed, BAP and PBAP chromatin remodeling complexes participate in differential regulation of *rho* expression [20], [24], [25]. Furthermore, the Chromodomain Helicase DNA-binding protein encoded by the TrxG gene *kismet* (*kis*) [26], [27] also plays a role in vein development [28]. *kis* mutants present ectopic wing veins, and increase ectopic vein phenotypes of mutants affecting the BAP complex component SNR1 [22], [24], [29]. Finally, loss of the ETP Corto [30], [31] induces ectopic veins and enhances ectopic vein phenotypes of several TrxG gene mutants [24], [32], implicating *corto* in control of vein *vs* intervein fate. Interestingly, Corto associates with ERK and its scaffold protein MP1 on chromatin, potentially linking ERK signaling to transcriptional regulation mediated by TrxG and ETPs [15], [32], [33].

During a two-hybrid screen using Corto as bait [34], we isolated Elongin C (EloC). This protein is a subunit of the Elongin complex, initially purified from rat liver extracts on its ability to increase the catalytic rate of RNA polymerase II (RNA-PolII) transcription *in vitro* and to suppress transient RNA-PolII pausing [35], [36]. The Elongin complex is constituted of three subunits, a catalytic subunit Elongin A (EloA), responsible for transcriptional activity, and two regulatory subunits Elongin B (EloB) and EloC, that can also participate in formation of an E3 ubiquitin ligase complex [37]–[40]. Whereas EloC increases EloA activity, EloB acts as a chaperon that facilitates Elongin complex assembly and enhances its stability [41]. The Elongin complex is evolutionarily conserved since EloA, B and C homologs are found in mammals, *C. elegans*, *D. melanogaster* and *S. cerevisiae* [35], [42]–[46]. In *Drosophila*, down-regulation of *EloA* by RNA interference causes lethality, which suggests that the Elongin complex is essential for development [46].

Here, we confirm the interaction between Corto and the three subunits of the Elongin complex both *in vitro* and *in vivo,* and we address the role of this complex during development, particularly in control of wing tissue fates. Using genetic analyses, we first demonstrate that the Elongin complex participates in wing tissue formation, and second that it antagonizes *corto* and several TrxG genes during this process. We show that EloC, like EloA, binds polytene chromosomes. Furthermore, EloC largely overlaps on chromatin with the epigenetic mark H3K36me3, which is associated with transcriptional elongation. Lastly, we report chromatin immunoprecipitation experiments showing that Corto and EloC bind the vein-promoting gene *rho* in wing imaginal discs. All these data suggest that Corto and the Elongin complex could participate in tissue-specific transcriptional regulation of *rho*.

## Materials and Methods

### *Drosophila* strains and genetic crosses

Flies were grown on standard yeast-cornmeal medium at 25°C, unless stated otherwise in the text. *w^1118^* was used as control strain. *corto^420^*, *corto^07128^*, *corto^L1^* and *UAS::FLAG-HA-CortoCD* lines were previously described [15], [31], [32]. Transgenic lines allowing Myc-EloC or FLAG-HA-EloC expression were generated by standard *P*-element mediated transformation, after cloning the *EloC* (*CG9291*) coding region into *pUASp::Myc* or *pUASp::FLAG-HA* vectors (Gateway™, gifts from Dr. T. Murphy, https://dgrc.cgb.indiana.edu/vectors). Lines *Ubx::flp; FRT82B, ubi-nlsGFP,* that induces recombination in all imaginal discs [47], and *hs::flp; act>CD2>Gal4, UAS::GFP*, used for flip-out experiments, were kindly provided by Drs. A. Audibert and J. Montagne, respectively. Lines *EloC^SH1520^* and *EloC^SH1299^* were from the Szeged Stock Center. All other lines, including drivers used to express *UAS-*driven transgenes ubiquitously (*daugtherless::Gal4*) or in wing imaginal discs (*scalloped::Gal4*, *Beadex::Gal4*, *spalt::Gal4*, *nubbin::Gal4, rotund::Gal4*, *C684::Gal4*, *OK10::Gal4*) were from the Bloomington Stock Center. Line *VALIUM20 EloC* (*ValEloC*) from the Transgenic RNAi Project (TRiP) at Harvard Medical School was used to down-regulate *EloC* by RNA interference (RNAi) [48]. In all crosses, wing phenotypes were analyzed in females, but arose also in males, although less frequently.

### Clonal analysis

Flip-out clones of cells in which *EloC* was down-regulated by RNAi were obtained by crossing *ValEloC* and *hs::flp, act>CD2>Gal4, UAS::GFP* flies. First instar larvae (24-48h AEL: After Egg Laying) were heat-shocked for 60 minutes at 37°C, and development was then resumed at 25°C. Clones of cells over-expressing *EloA* were obtained by crossing *Ubx::flp* and *act>CD2>Gal4, UAS::GFP*; *EloA^G4930^* flies. Clones of homozygous *corto^420^* cells were obtained by crossing *Ubx::flp; FRT82B, ubi-nlsGFP* and *FRT82B, corto^420^* flies. Third instar larval progeny from these crosses were dissected, fixed for 20 minutes in PBS with 3.7% paraformaldehyde and stained with DAPI. Wing imaginal discs were mounted in Mowiol and visualized by fluorescent microscopy (Nikon Eclipse 80i microscope).

### RT-qPCR experiments

RT-qPCR experiments were carried out in a CFX96™ system (Biorad) using SsoFast EvaGreen™ Supermix (Biorad). cDNA were synthesized from total RNA extracted from 0–24h embryos or third instar larvae, as previously described [15], and quantified using the standard curve method, with *Rp49*, *RpL12* or *eIF-2α* for normalization. Primers were:

EloA-F, 5′-GTGGAATCAGACTGCTGCTGTCG-3′

EloA-R, 5′-CGACCCAGCGGGATGCAACA-3′

EloB-F, 5′-CCGAGCTGAAGCGAATGATTGAG-3′

EloB-R, 5′-GTGGACACCGTCACGCCGTA-3′

EloC-F, 5′-TCGTCAAACGCGAGCACGCT-3′

EloC-R, 5′-GGCAAACTGACCCGGTCCGG-3′

Rp49-F, 5′-CCGCTTCAAGGGACAGTATC-3′

Rp49-R, 5′-GACAATCTCCTTGCGCTTCT-3′

RpL12-F, 5′-CCTCCCAAATTCGACCCAA-3′

RpL12-R, 5′-CACGCAACGCAGGTACACC-3′

eIF-2α-F, 5′- TCGCATCAACCTGATAGCAC-3′

eIF-2αR, 5′- ATCGTACTCGCTGGTCTTGG-3′

### S2 cell transfection and co-immunoprecipitation

*EloA* (*CG6755*), *EloB* (*CG4204*), *EloC* (*CG9291*) and *corto* (*CG2530*) cDNA were cloned into Gateway™ *Drosophila* vectors (gifts from Dr. T. Murphy, https://dgrc.cgb. indiana.edu/vectors) to express either Myc- or FLAG-tagged fusion proteins under control of the *actin5C* promoter. These vectors were transiently transfected into S2 cells and total protein extracts were prepared as previously described [33]. For cross-linking, cells were treated with paraformaldehyde (1%) for 10 minutes at room temperature prior to protein extraction. Co-immunoprecipitation experiments were carried out as previously described, using anti-FLAG (F3165, Sigma) or anti-Myc (sc-40, Santa Cruz Biotechnology) antibodies [15].

### Immunostaining of polytene chromosomes

Co-immunostainings of polytene chromosomes from *da::Gal4>>Myc-EloC* larval salivary glands were performed as previously described [30], using mouse anti-Myc (1:60) (sc-40, Santa Cruz Biotechnology), rabbit anti-H3K36me3 (1:60) (pAB-058-050, Diagenode) or rabbit anti-Corto (1:20) [30] antibodies. Secondary antibodies (Alexa™ Fluor 488 anti-mouse IgG and Alexa™ Fluor 594 anti-rabbit IgG, Molecular Probe) were used at 1:1000 dilution.

### Chromatin immunoprecipitation (ChIP)

ChIP experiments were performed using chromatin from *sd::Gal4>>UAS::FLAG-HA-EloC* or *sd::Gal4>>UAS::FLAG-HA-CortoCD* third instar larval wing discs fixed with paraformaldehyde. The protocol, modified from Pérez-Lluch *et al*., was described previously [31], [49]. Immunoprecipitated and input DNAs were purified in 70 µl of water with IPure kit following the manufacturer's instructions (Diagenode). q-PCR reactions were performed on 5 µl of DNA in a CFX96™ system (Biorad) using SsoFast EvaGreen™ Supermix (Biorad). q-PCR data were normalized against input sample and expressed as percentages of input. Primers located in *rhomboid* (*rho*) or in an untranscribed region of *Scr* regulatory sequences not bound by Corto in embryos and S2 cells [50] and used as a negative control (NC) were:

rho-165F, 5′-TGTGGCAAGGCGGCAGATGG-3′

rho-165R, 5′ –TGTGTGGGTGGGTGGGGTGT-3′

rho+51F, 5′-AGTCAGTTGCGTGCGAGCCG-3′

rho+51R, 5′-CAGTCCGACTTTCTCAGTTTGA-3′

rho+2812F, 5′-CGTCGGATTCGGTGCTGGTC-3′

rho+2812R, 5′-GGTGGAACCAGTTGGCGTGC-3′

NC-F, 5′-GGCAGCTGTTCAAATCGGAGGCT-3′

NC-R, 5′-TCACGTCGAGGTGTTCGGCG-3′

## Results

### Corto interacts with the three subunits of the Elongin complex *in vivo*

**Figure 1:**
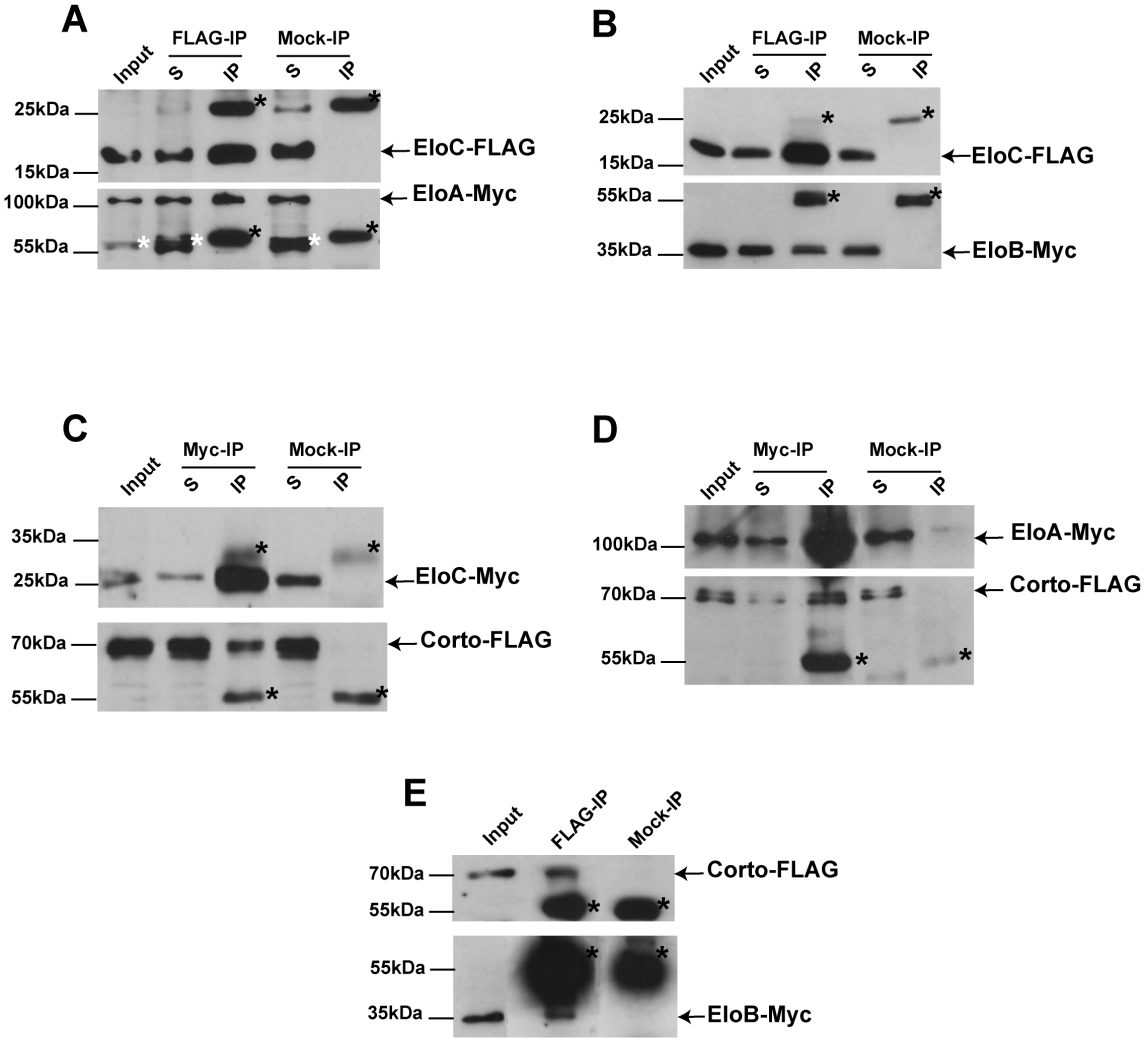
Corto interacts with the Elongin complex *in vivo.* (A, B): EloA-Myc (A) and EloB-Myc (B) co-immunoprecipitate with EloC-FLAG; (C, D): Corto-FLAG co-immunoprecipitates with EloC-Myc (C) and EloA-Myc (D); (E): EloB-Myc co-immunoprecipitates with Corto-FLAG. In D and E, co-immunoprecipitations were performed after cross-linking. In E, exposure time for the lower panel was 50 times longer than for the upper panel. Immunoprecipitations were performed with anti-Myc (Myc-IP), anti-FLAG (FLAG-IP) or anti-HA (Mock-IP) antibodies. Immunoprecipitated proteins were revealed by Western blot using anti-FLAG or anti-Myc antibodies. Arrows show immunoprecipitated proteins, and black asterisks point to heavy or light IgG chains. In A, white asterisks indicate a non-specific band. S: supernatant after immunoprecipitation; IP: protein G-agarose beads. 5% of the input or supernatant and 50% of the immunoprecipitate were loaded onto the gels.

To validate the interaction between Corto and EloC detected in a two-hybrid screen [34], and to test whether Corto interacted also with the EloA catalytic and EloB regulatory subunits of the Elongin complex, we performed co-immunoprecipitation experiments with Myc- and FLAG-tagged proteins expressed in *Drosophila* S2 cells. We first tested co-immunoprecipitation between the three tagged Elongin proteins. These were mainly detected in nuclear extracts of S2 cells (data not shown). Using whole cell extracts, we detected a strong interaction between EloA and EloC (Figure 1A), as well as between EloB and EloC (Figure 1B). By contrast, EloA and EloB co-immunoprecipitated very weakly and only after cross-linking with paraformaldehyde (data not shown).

We next addressed interactions between the three Elo proteins and Corto. We observed strong co-immunoprecipitation between EloC and Corto (Figure 1C). No interaction between Corto and EloA or EloB was detected when using native protein extracts. However after paraformaldehyde cross-linking, we observed strong co-immunoprecipitation between Corto and EloA (Figure 1D), but only a very weak one between Corto and EloB (Figure 1E). These results confirm the two-hybrid interaction between Corto and EloC. In addition, they suggest that Corto interacts with the whole Elongin complex, probably *via* direct binding to EloC.

### EloC binds polytene chromosomes

**Figure 2:**
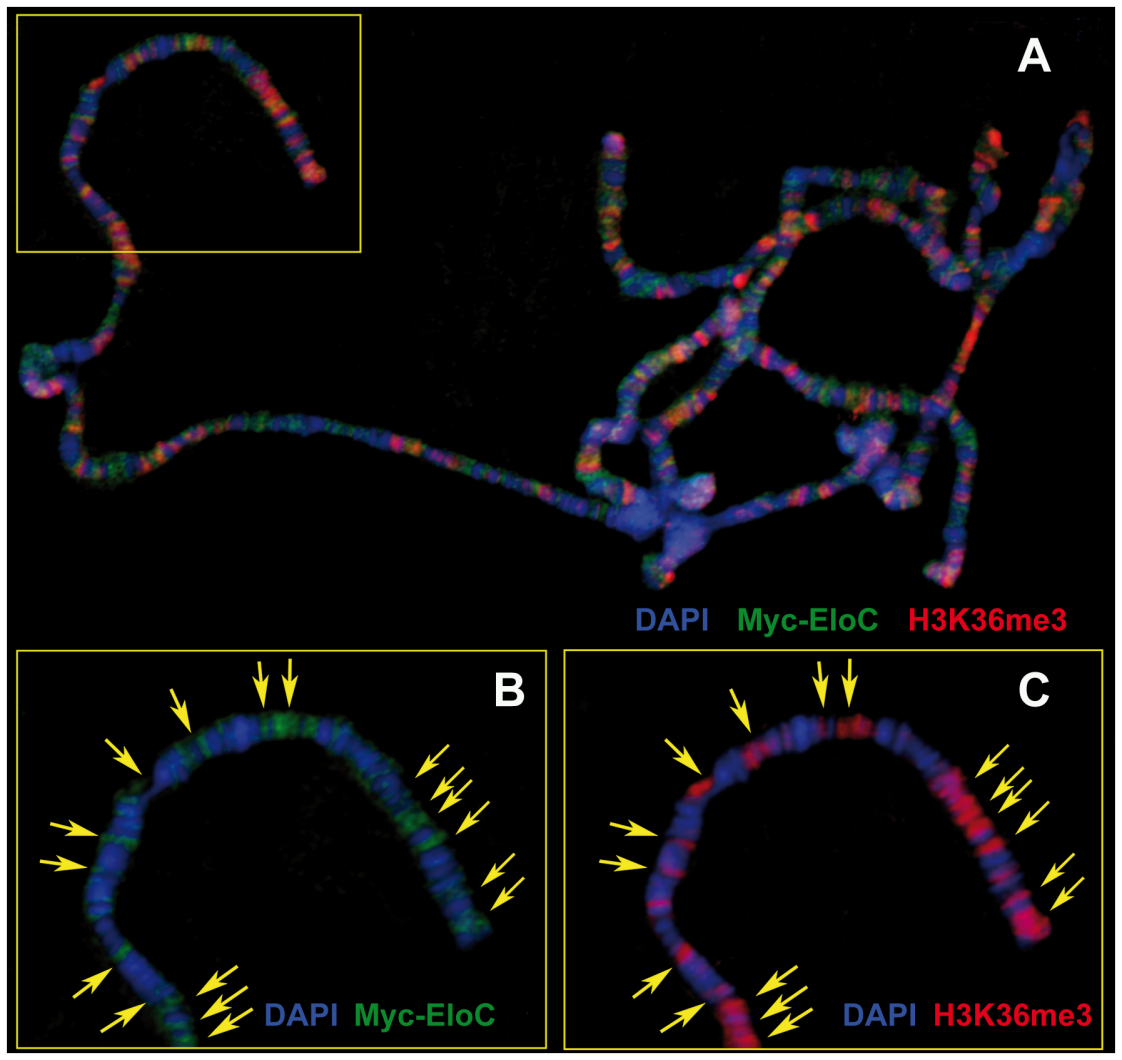
EloC binds polytene chromosomes. (A): Polytene chromosomes from salivary glands of *da::Gal4>>Myc-EloC* larva (two sets of chromosomes are shown). Myc-EloC (green) exhibits many co-localizations with H3K36me3 (red). (B, C): Close-up views of the region framed by a yellow rectangle in A. Yellow arrows on B and C indicate co-localizations between Myc-EloC and H3K36me3. DNA was stained with DAPI (blue).

We next addressed whether EloC, like EloA and Corto, could bind polytene chromosomes. *Drosophila* EloA binds polytene chromosomes at many sites and extensively co-localizes with RNA-PolII phosphorylated on serine 5 (RNA-PolII-S5p, paused form) as well as on serine 2 (RNA-PolII-S2p, elongating form) [46], [51]. However, overlap between EloA and phospho-RNA-PolII was not complete, suggesting that the Elongin complex may not have a general role in transcription, but rather act as a transcriptional activator for a subset of genes. To analyze global EloC binding to chromatin, we generated a line containing a *UASp-Myc-EloC* transgene. Immunostainings of polytene chromosomes from larvae expressing this transgene driven by the ubiquitous *daugtherless::Gal4* (*da::Gal4*) driver showed that Myc-EloC bound polytene chromosomes at many sites. These were preferentially located at DAPI interbands, suggesting that Myc-EloC localized to open chromatin (Figure 2A, B). When we co-immunostained polytene chromosomes from *da::Gal4>>Myc-EloC* larval salivary glands with antibodies against Myc and Corto, only a few common sites were observed (data not shown). Interestingly, Myc-EloC extensively co-localized with H3K36me3, an epigenetic mark that correlates with transcriptional elongation [52], [53] (Figure 2A-C). Together these data show that EloC, like EloA, binds chromatin protein and preferentially localizes to transcriptionally active sites.

### *EloB* and *EloC* are essential genes

**Figure 3:**
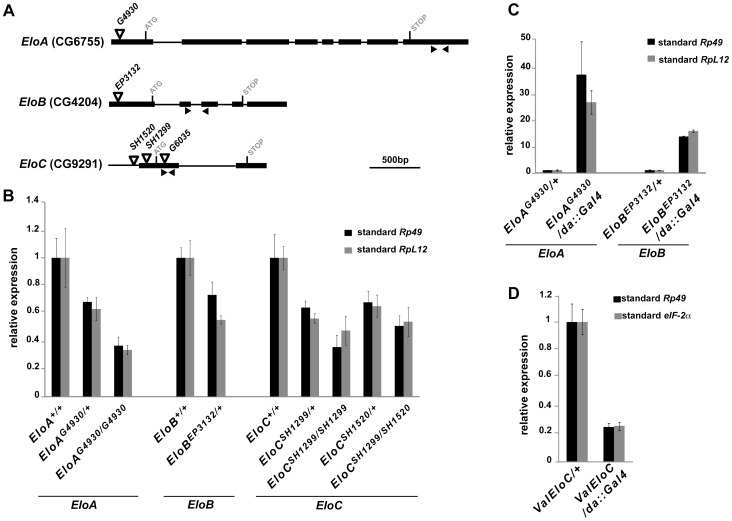
Deregulation of *EloA, EloB* or *EloC* expression using *P*-element insertion lines. (A): Structure of *EloA*, *EloB* and *EloC* genes showing localization of the *P*-elements used in this study. Exons are represented by boxes, and introns by lines. Black arrowheads show positions of primer pairs used to quantify *Elo* gene expression. (B): Quantification of *Elo* gene expression in *EloA^G4930^*, *EloB^EP3132^*, *EloC^SH1520^* or *EloC^SH1299^* homozygous or heterozygous larvae. (C): Quantification of *Elo* gene expression in *da::Gal4>>EloA^G4930^* or *da::Gal4>>EloB^EP3132^* larvae. (D): Quantification of *EloC* expression in *da::Gal4*>>*ValEloC* embryos. Relative *Elo* expression levels were obtained by normalization to *Rp49* (black bars, B to D), *RpL12* (grey bars, B, C) or *eIF-2α* (grey bars, D).

No mutations for *EloA, B* or *C* being reported so far, we analyzed transgenic lines carrying a *P*-element in or close to *Elo* genes (Figure 3A). Line *EloA^G4930^* has a *UAS-*containing *P*-element located 58bp downstream of the *EloA* transcription start site (TSS), potentially allowing *EloA* over-expression with a *Gal4* driver [54]. Line *EloB^EP3132^* contains a similar element inserted 21bp downstream of the *EloB* TSS [54], [55]. Lines *EloC^SH1520^* and *EloC^SH1299^* have *P-*elements 31bp upstream and 34bp downstream of the *EloC* TSS, respectively [56]. To determine whether these insertions impaired *Elo* gene transcription, we quantified *Elo* mRNA levels in heterozygous or homozygous third instar larvae. As shown in Figure 3B, all four insertions decreased expression of the corresponding *Elo* gene (1.4 to 3-fold reduction) and behaved thus as hypomorphic, loss-of-function *Elo* alleles. Moreover, *EloA^G4930^* and *EloB^EP3132^* driven ubiquitously with *da::Gal4* induced high over-expression of the corresponding gene (35- and 15-fold increase, respectively) (Figure 3C). We also tested a *VALIUM20* transgenic line (*ValEloC*) that allows *EloC* down-regulation by RNA interference (RNAi) [48]. Ubiquitously driven in embryos with *da::Gal4*, *ValEloC* induced strong *EloC* down-regulation (Figure 3D, 5-fold reduction). Altogether, these lines allowed us to genetically address the functions of *Elo* genes.

Down-regulation of *EloA* by RNAi induces lethality during the pupal stage [46], indicating that *EloA* is an essential gene. *EloA^G4930^* homozygotes, on the other hand, are viable, which suggests that *EloA^G4930^* individuals produce enough protein to correctly achieve development, despite the decreased level of *EloA* mRNA. Homozygous *EloB^EP3132^* larvae died before the third larval instar. Furthermore, when associating *EloB^EP3132^* with a deficiency uncovering *EloB* (*Df(3R)BSC518*), only one *EloB^EP3132^/Df(3R)BSC518* adult escaper hatched among 272 balanced progeny. Together, these results indicate that *EloB* loss-of-function is either subviable or lethal.

To address EloC function, we used lines *EloC^SH1520^* and *EloC^SH1299^* as well as line *EloC^G6035^*, obtained independently of the two others. *EloC^G6035^* line carries a *P*-element in *EloC* coding sequence 157bp downstream of the ATG [54], and could thus be a null allele of *EloC*. All three insertions were homozygous lethal. Lethality occurred before the third larval instar for *EloC^SH1520^* and *EloC^G6035^*, and during this instar for *EloC^SH1299^*. Since no deficiency including *EloC* was available, we examined viability of heteroallelic *EloC* animals combining *EloC* alleles two by two. None of the three trans-allelic combinations gave viable adults. The rare *EloC^SH1520^/EloC^SH1299^* third instar larvae died before pupariation. Furthermore, ubiquitous RNAi-mediated down-regulation of *EloC* (*da::Gal4>>ValEloC*) induced complete embryonic lethality. Altogether, these results show that *EloC* is an essential gene. We also addressed whether over-expression of *Elo* genes would affect viability. Ubiquitous over-expression of *EloA*, *EloB* or *EloC* was performed driving *EloA^G4930^*, *EloB^EP3132^* or *UAS::Myc-EloC* with *da::Gal4. N*one of these over-expressions affected fly viability.

In conclusion, these results showed that the EloB and EloC regulatory subunits of the Elongin complex are essential for development, like the catalytic EloA subunit.

### EloC is required during wing development

**Figure 4:**
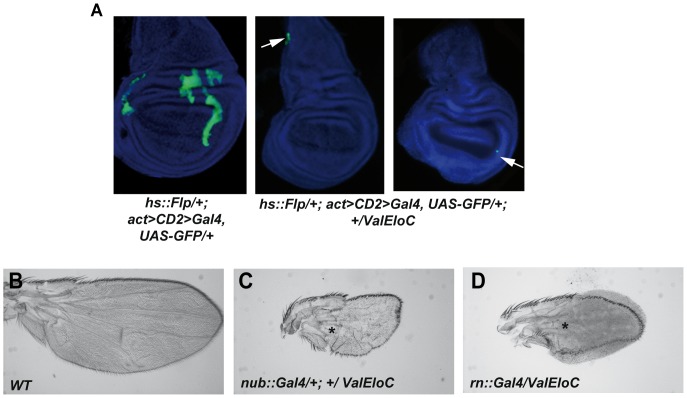
Down-regulation of *EloC* by RNA interference impairs both cell proliferation and cell differentiation in wing imaginal discs. (A): Clones expressing the *ValEloC* transgene (GFP^+^ cells, shown by white arrows) are located at the periphery of the disc and are very small compared to control clones. (B, C, D): Wings from pharates in which *ValEloC* is driven by *nub::Gal4* (C) or *rn::Gal4* (D), both expressed in the wing pouch [66], [67] are small compared to wild-type pharate wings (B) and exhibit severe wing blade defects. By contrast, longitudinal veins (shown by asterisks) are formed in the proximal-most part of the wing blade where *nub::Gal4* and *rn::Gal4* are not expressed.

To better understand the need for EloC during wing development, we generated flip-out clones of cells expressing *ValEloC,* using a flipase gene under control of a heat-shock promoter (*hs::flp; act>CD2>Gal4, UAS::GFP*). Comparing the frequencies of late third instar wing discs with either *ValEloC* or control clones, we found that the former tended to be less frequent than the latter (*ValEloC*: 5 out of 34 discs; control: 11 out of 32 discs). *ValEloC* clones were much smaller than control clones, and always located at the disc periphery (Figure 4A). In addition, no adult wing phenotype was observed. This result thus shows that most of the clonal *ValEloC* cells induced in the wing pouch during the first larval instar either stopped dividing, died or were eliminated from the disc before the late third instar, suggesting that *EloC* is important for cell viability.

We next associated *ValEloC* with various wing disc drivers expressed at different developmental stages (*scalloped::Gal4*, *spalt::Gal4*, *nubbin::Gal4, rotund::Gal4*, *C684::Gal4*, *OK10::Gal4*). When grown at 25°C, the progeny of all these crosses died as third instar larvae or pupae, depending on the driver. At 20°C, where the UAS/Gal4 system is less active, escaper adults were obtained only with the *nubbin* (*nub::Gal4*) and *rotund* (*rn::Gal4*) drivers. Both drivers are specifically expressed in the wing pouch, and *rn::Gal4* is expressed only from the third larval instar onwards. The escaper flies presented tiny misshapen wings with severe growth and differentiation defects in the area of driver expression (Figure 4B-D). Hence, even late wing pouch specific down-regulation of *EloC* has drastic consequences on *Drosophila* wing development. Altogether, these data show that decrease in *EloC* expression impedes cell growth and/or proliferation, stressing the importance of *EloC* during wing development.

### The three Elongin complex subunits participate in control of wing cell identity

**Figure 5:**
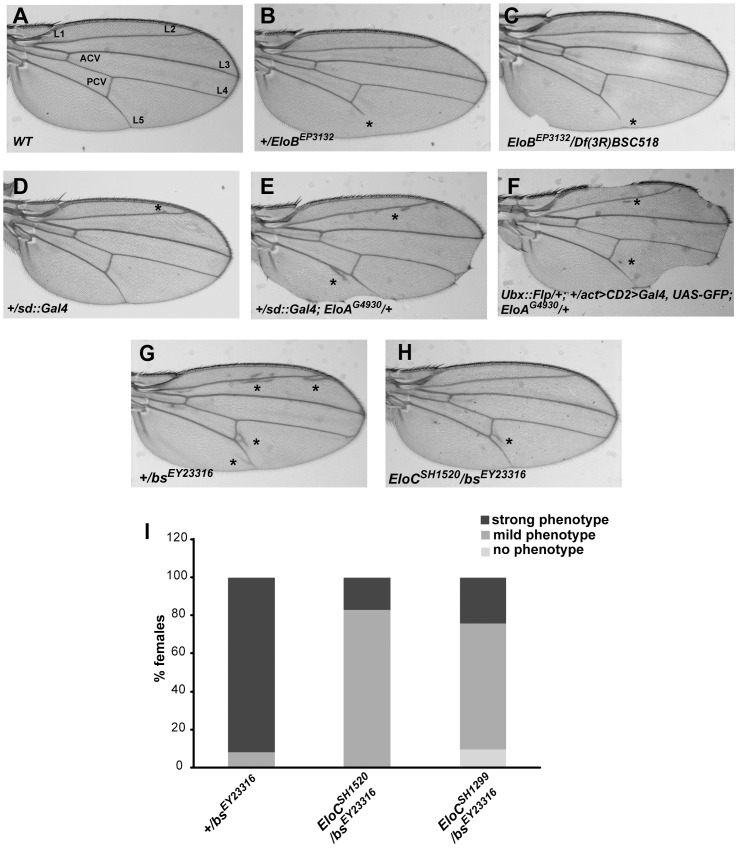
*Elo* genes control wing cell identity. (A): Wing from control *w^1118^* fly (L1-L5: longitudinal veins; ACV and PCV: anterior and posterior cross-veins). (B, C): Wings from *+/EloB^EP3132^* and *EloB^EP3132^/Df(3R)BSC518* flies exhibit truncated L5. (D): Wings from *+/sd::Gal4* flies have a very faint ectopic vein phenotype and no margin phenotype. (E, F): Wings from flies over-expressing *EloA* exhibit ectopic vein and margin phenotypes. (G, H, I): *EloC^SH1520^* and *EloC^SH1299^* loss-of-function alleles diminish expressivity of the ectopic vein phenotype induced by the *bs^EY23316^* loss-of-function allele. Strong phenotype: ectopic veins everywhere in the wing (shown in G). Mild phenotype: ectopic veins under the posterior cross-vein only (shown in H).

Among heterozygous *EloB^EP3132^* females, 28.8% showed a truncated L5 vein (Table 1, Figure 5B). *EloC* heterozygous females presented the same phenotype, although less penetrant (Table 1, *EloC^SH1520^* and *EloC^G6035^*, 1.4% and 1.1%, respectively), as did the single *EloB^EP3132^/Df(3R)BSC518* escaper (Figure 5C). Homozygous *EloA^G4930^* flies, on the other hand, had normal wings. To further analyze links between the Elongin complex and wing morphogenesis, we over-expressed *Elo* genes in wing imaginal discs. No wing phenotype was observed when over-expressing *EloB* or *EloC,* whereas *EloA* over-expression consistently affected wing morphogenesis. Driven by *scalloped::Gal4* (*sd::Gal4)*, *EloA* significantly increased ectopic veins compared to heterozygous *sd::Gal4* controls (53.7% *vs* 33% of females) (Table 1; Figure 5D, E), and induced margin defects. Ectopic veins were also significantly enhanced when driving *EloA* with the wing-specific *Beadex::Gal4* (*Bx::Gal4*) line (85.5% *vs* 16.5% of control females) (Table 1). Lastly, wings with *EloA* over-expressing clones (induced with *Ubx::flp*) presented both margin defects and ectopic veins (Figure 5F). Taken together, these data show that increased *EloA* expression caused margin and vein defects. Interestingly, the *EloA* over-expression phenotype (presence of ectopic veins) was opposite to the *EloB* and *EloC* loss-of-function phenotype (vein truncation).

**Table 1:** *EloA and EloC* deregulation induce wing vein defects.

Genotype	Number of females observed	Vein phenotype observed	% females with vein phenotype
*+/EloB^EP3132^*	170	Truncated L5	28.8
*EloC^SH1520^/+*	143	Truncated L5	1.4
*EloC^G6035^/+*	175	Truncated L5	1.1
*+/sd::Gal4*	72	Ectopic vein	33
*+/sd::Gal4; EloA^G4930^/+*	121	Ectopic vein	53.7^ a^
*Bx::Gal4/+*	133	Ectopic vein	16.5
*Bx::Gal4/+; +/EloA^G4930^*	139	Ectopic vein	85.5^ a^

The upper allele was brought by the mother. Numbers of *+/sd::Gal4;EloA^G4930^/+* or *Bx::Gal4/+;+/EloA^G4930^* females with ectopic veins were compared to numbers of *+/sd::Gal4* or *Bx::Gal4/+* females with ectopic veins, respectively (z-test, ^a^ p<0.001).

To further clarify the link between the subunits of the Elongin complex and specification of wing tissue fate, we combined *EloC* alleles with a loss-of-function allele of *blistered* (*bs*), *bs^EY23316^. bs* is required for intervein tissue formation [10], [11]. All heterozygous *bs^EY23316^* flies presented ectopic veins, although with different expressivity (Table 2; Figure 5G). Interestingly, *EloC^SH1520^ and EloC^SH1299^* alleles both strongly reduced expressivity and penetrance of the *bs^EY23316^* ectopic vein phenotype. Indeed, significantly less flies presented the strongest phenotype (17% for *EloC^SH1520^/bs^EY23316^* females and 24.2% for *EloC^SH1299^/bs^EY23316^* females *vs* 92% for control *+/bs^EY23316^* females), and 9.5% of *EloC^SH1299^/bs^EY23316^* flies had no ectopic veins (Table 2; Figure 5H, I).

**Table 2:** Decreasing *EloC* expression suppresses ectopic veins induced by *blistered* loss-of-function.

Genotype	Number of females observed	% females with no ectopic vein	% females with mild ectopic vein phenotype	% females with strong ectopic vein phenotype
*+/bs^EY23316^*	125	0	8	92
*EloC^SH1520^/bs^EY23316^*	112	0	83^ a^	17^ a^
*EloC^SH1299^/bs^EY23316^*	95	9.5^ a^	66.3^ a^	24.2^ a^

The upper allele was brought by the mother. The number of *EloC*/*bs^EY23316^* females with ectopic veins was compared to the number of *+/bs^EY23316^* females with ectopic veins (z-test, ^a^ p<0.001). The mild ectopic vein phenotype corresponds to presence of ectopic veins distal to the posterior cross-vein (Figure 5H), whereas the strong ectopic vein phenotype corresponds to presence of ectopic veins everywhere in the wing (Figure 5G).

Altogether, these results suggest that the three subunits of the Elongin complex participate in specification of wing tissue fate, playing a vein-promoting role.

### *Elo* mutations antagonize wing phenotypes of *corto* and TrxG mutants

**Figure 6:**
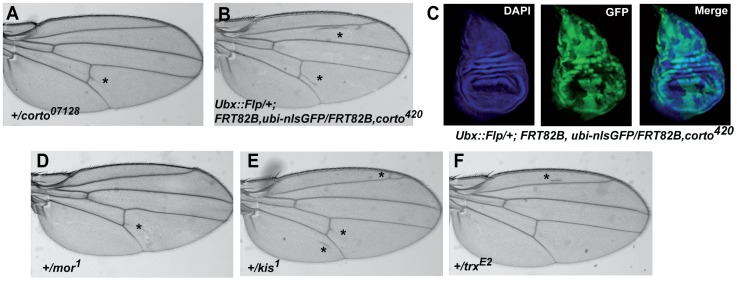
*corto* and several TrxG genes control wing cell identity. (A, B): Ectopic vein phenotypes induced by the *corto^07128^* loss-of-function allele (A) or by *corto^420^* loss-of-function clones (B). (C): *corto^420^* homozygous clones (GFP^-^ cells) in wing imaginal discs. (D, E, F): Ectopic vein phenotypes induced by *mor*, *kis* or *trx* loss-of-function alleles. In A, B, D, E, F, asterisks mark ectopic veins.

To understand the functional relationships between *Elo* genes and *corto*, we analyzed their genetic interactions. As previously reported, heterozygous *corto* loss-of-function flies as well as the rare heteroallelic *corto* escapers exhibited ectopic veins [15] (Table 3; Figure 6A). We found that ectopic veins were also observed when homozygous *corto^420^* clones were induced in wing imaginal discs (Figure 6B). Strikingly, although clones were distributed all over the disc (GFP^-^ cells, Figure 6C), ectopic veins preferentially formed close to longitudinal veins 2 and 5, and to the posterior cross-vein. A similar observation has been made for mutants of several other genes involved in wing tissue formation [5], [57]. When combining *corto* loss-of-function alleles with mutant alleles for each of the three *Elo* genes, ectopic veins were significantly reduced, even by the homozygous viable *EloA^G4930^* allele (Table 3). Hence, all three *Elo* genes counteract *corto* in vein identity specification.

**Table 3:** Decreasing *EloA, EloB* or *EloC* expression partially suppresses ectopic veins induced by *corto* loss-of-function.

Genotype	Number of females observed	% females with ectopic veins
*+/corto^07128^*	96	93.8
*corto^07128^/+*	78	97.4
*corto^07128^/EloA^G4930^*	183	38.3^ a^
*corto^07128^/EloB^EP3132^*	128	26.6^ a^
*EloC^SH1520^/+; +/corto^07128^*	156	14.8^ a^
*EloC^SH1299^/+; +/corto^07128^*	69	27.5^ a^
*+/corto^L1^*	164	40.2
*corto^L1^/+*	51	49
*corto^L1^/EloA^G4930^*	191	0^ a^
*corto^L1^/EloB^EP3132^*	129	0.8^ a^
*EloC^SH1520^/+; +/corto^L1^*	119	0^ a^
*EloC^SH1299^/+; +/corto^L1^*	114	0^ a^

The upper allele was brought by the mother. The number of females with ectopic veins among flies transheterozygous for *Elo* and *corto* mutations was compared to the number of females with ectopic veins among flies with a *corto* mutation only (z-test, ^a^ p<0.001).

We next looked for genetic interactions between *Elo* genes and the TrxG genes *moira* (*mor*), *kismet* (*kis*) and *trithorax* (*trx*) that are implicated in wing tissue formation and interact with *corto* in this process [24], [32], [58]. We confirmed that wings of flies heterozygous for *mor^1^* or *kis^1^* loss-of-function alleles presented ectopic veins (Table 4; Figure 6D, E) [28], [59]. Ectopic veins were also observed in flies heterozygous for the *trx^E2^* loss-of-function allele (Table 4; Figure 6F). As observed for *corto*, ectopic veins were significantly reduced when combining these TrxG alleles with mutant alleles for each of the three *Elo* genes (Table 4). Taken together, these genetic interactions indicate that the three *Elo* genes promote vein cell identity in the wing disc. Furthermore, they counteract *corto* and several TrxG genes for vein *vs* intervein cell identity.

**Table 4:** Decreasing *EloA* or *EloC* expression partially suppresses ectopic veins induced by TrxG gene loss-of-function.

Genotype	Number of females observed	% females with ectopic veins
*+/mor^1^*	93	22.5
*EloA^G4930^/mor^1^*	110	5.5^ a^
*EloC^SH1520^/+; +/mor^1^*	132	1.5^ a^
*EloC^SH1299^/+; +/mor^1^*	78	6.4^ a^
*+/kis^1^*	81	81.5
*+/kis^1^; EloA^G4930^/+*	94	48.8^ a^
*EloC^SH1520^/kis^1^*	80	17.5^ a^
*EloC^SH1299^/kis^1^*	54	9.2^ a^
*+/trx^E2^*	89	53.9
*EloA^G4930^/trx^E2^*	96	10.4^ a^
*EloC^SH1520^/+; +/trx^E2^*	93	5.4^ a^
*EloC^SH1299^/+; +/trx^E2^*	54	16.7^ a^

The upper allele was brought by the mother. Numbers of females with ectopic veins among flies transheterozygous for *Elo* and TrxG mutations were compared to numbers of females with ectopic veins among flies with a TrxG mutation only (z-test,^a^ p<0.001).

### The vein promoting gene *rhomboid* (*rho*) could be a common target of Corto and the Elongin complex

**Figure 7:**
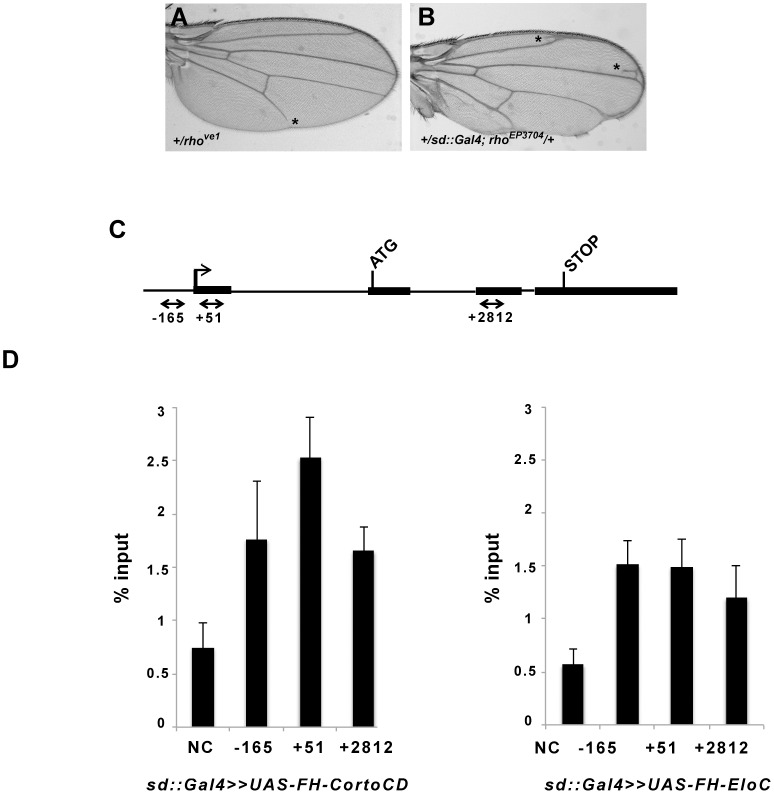
EloC and Corto bind *rho* in wing imaginal discs. (A, B): Wing phenotypes induced by *rho* loss-of-function (A) or over-expression (B). Asterisks mark truncated L5 (in A) or ectopic veins (in B). (C): Schematic structure of *rho* with exons represented by boxes and introns by lines. Black arrows show primer pairs used for ChIP experiments. (D): Binding of Corto chromodomain (FH-CortoCD) and EloC (FH-EloC) on *rho*. For each genotype, the mean of two independent experiments is shown. Error bars correspond to standard deviations.

L5 vein truncation, observed in *EloB* and *EloC* heterozygous mutants, was also observed in mutants for the vein-promoting gene *rhomboid* (*rho*): 13.2% (11/83) of female heterozygotes for the hypomorphic *rho^ve1^* allele [5] and 24.2% (39/161) of female heterozygotes for the amorphic *rho^7M43^* allele [55] presented a truncated L5 vein (Figure 7A). Furthermore, as previously described [15], over-expression of *rho* (*sd::Gal4>*>*rho^EP3704^*) induced ectopic vein and margin phenotypes recalling the ones induced by *EloA* over-expression (compare Figures 7B and 5E). These observations led us to hypothesize that the Elongin complex could directly activate *rho* expression. On the other hand, wings of *corto* loss-of-function pupae exhibit ectopic expression of *rho* in intervein tissue [15]. Corto could thus be involved in direct repression of *rho* in future intervein cells. Therefore, Corto and the Elongin complex could act in opposite ways on *rho* regulation, an hypothesis in agreement with our genetic data showing that *Elo* genes antagonize the role of *corto* in vein *vs* intervein cell identity.

To address direct regulation of *rho* by Corto and the Elongin complex, we analyzed the binding of Corto and EloC to *rho* in late third instar wing imaginal discs by chromatin immunoprecipitation. We used the *sd::Gal4* driver to express FLAG-HA (FH) tagged forms of either EloC (FH-EloC) or the Corto chromodomain (FH-CortoCD), that was previously shown to mimic Corto binding to polytene chromosomes [31]. Very few ectopic veins (due to the driver) were observed in wings of *sd::Gal4>>FH-EloC* flies or *sd::gal4>>FH-CortoCD* flies (data not shown), suggesting that the pattern of *rho* expression in these genetic contexts was similar to the one of wild-type wing imaginal discs. We found that FH-EloC as well as FH-CortoCD bound *rho*, the latter being slightly enriched just after the *rho* TSS (Figure 7C, D). In third instar larva wing imaginal discs, *rho* expression is restricted to the few cells that will give rise to the future veins and wing margin [5].The large majority of wing disc cells are thus future intervein cells in which *rho* is repressed. Hence, these results suggest that, in intervein cells, the Elongin complex and Corto are able to bind *rho*.

## Discussion

### The ETP Corto interacts with the three subunits of the elongation complex Elongin *in vivo*

We show here that, in *Drosophila* as in mammals [40], the three Elongin proteins Elo A, B, and C are mainly nuclear and interact two by two. EloC/B and EloC/A interactions may be direct, as they were observed without cross-linking treatment. By contrast, EloA/B interaction is more labile and may thus be indirect. It is possible that *Drosophila* EloC mediates the interaction between EloA and EloB, as previously shown in mammals [41], [60]. We also show that the ETP Corto interacts with all three Elo proteins, suggesting that Corto interacts with the Elongin complex. Hence, Corto and the Elongin Complex could share transcriptional targets. Several studies have shown that EloC binds its partners through a degenerate BC box motif, defined as (L,M)XXX(C,S)XXX(Í) [40], [44]. Two putative BC boxes (aa 357–365 and aa 542–550) are present in the C-terminal part of Corto. However, deletion of these sequences did not impair co-immunoprecipitation between Corto and EloC (data not shown), suggesting that these two proteins interact through another unidentified sequence.

### The three Elongin complex subunits are essential for development

We present here the first characterization of lines allowing deregulation of *EloB* or *EloC* expression. *EloB* or *EloC* loss-of-function mutations induce early lethality (before the third larval instar), demonstrating that EloB and EloC, like EloA [46], are essential proteins. Our clonal and tissue-specific analyses of *EloC* mutant cells reveal that EloC is critically required all through wing development. By contrast, RNAi-mediated *EloA* down-regulation only induced lethality during the pupal stage [46], indicating either a less efficient reduction of *EloA* mRNA or a longer perdurance of maternal EloA. Alternatively, requirement of EloB and EloC in other complexes, such as an E3 ubiquitin ligase complex [37]–[40], might explain this difference.

### The three subunits of the Elongin complex participate in determination of wing cell identity

*EloB*/*C* loss-of-function as well as *EloA* over-expression induced wing phenotypes, mostly vein phenotypes. Interestingly, these loss-of-function and over-expression phenotypes are opposite (*i.e* truncated L5 vein for loss-of-function, ectopic veins for over-expression). Furthermore, whereas *EloA* over-expression induced ectopic veins, no phenotype was observed when over-expressing *EloB* and *EloC*. This result suggests that the amount of catalytic subunit EloA might be critical for Elongin complex function. In mammals, EloA is indeed the limiting component of the Elongin complex, EloB and EloC being in large excess (100 to 1000-fold more abundant than EloA) [40], [61]. Curiously, a previous study reported that mitotic clones for a deficiency that uncovers *EloA*, produced ectopic wing veins [62]. As this deletion uncovers more than 10 genes that may influence vein formation, we favor the hypothesis, in agreement with all data presented above, that *EloA* loss- of-function leads to loss of vein tissue. Alternatively, EloB and EloC, which also belong to ubiquitin ligase complexes, might modulate vein *vs* intervein cell fate in this context.

Altogether, our observations suggest that the Elongin A, B, C subunits promote vein cell identity. On the opposite, Corto maintains intervein cell identity, possibly *via* interaction with TrxG complexes. As Corto and EloC co-localize at a few sites on polytene chromosomes, they might have common transcriptional targets. A balance between Corto and the Elongin complex might fine-tune transcription of such genes.

### The vein-promoting gene *rho* could be a common target of Corto and the Elongin complex

In *corto* mutants, we previously showed that ectopic veins perfectly match with ectopic expression of *rho,* the first vein-promoting gene to be expressed [15]. As *Elo* gene mutations counteract *corto* mutations during formation of ectopic veins, we propose that *rho* could be a common target of Corto and the Elongin complex in intervein cells. In agreement with this hypothesis, immunoprecipitation using chromatin from late third instar wing imaginal discs, that can be assimilated to chromatin of intervein cells, revealed the presence of both Corto and EloC on *rho*. Two independent genome-wide studies on whole embryos and embryonic S2 cells have shown that poised RNA-PolII binds the *rho* promoter, suggesting that *rho* expression is controlled by “pause and release” of the transcriptional machinery [63], [64]. Interestingly, we found that Corto is slightly enriched just after the *rho* TSS, a position usually occupied by paused RNA-PolII (for a review, see [65]). Corto shares many sites on polytene chromosomes with paused RNA-PolII-S5p, suggesting that it is involved in transcriptional pausing [31]. On the other hand, we found that EloC co-localizes with H3K36me3, that characterizes transcriptional elongation, and the Elongin complex was shown to suppress transient RNA-PolII pausing [35], [36]. Hence, in future intervein cells, Corto and the Elongin complex could apply opposite forces on the transcriptional machinery at the *rho* promoter. Corto would block *rho* transcription whereas the Elongin complex would be ready to accompany *rho* elongation if release should occur. In future vein cells on the other hand, the Elongin complex could actively participate in *rho* transcriptional elongation, since loss of function mutants for *EloB* and *EloC* exhibit loss of vein tissue. In these cells, *rho* expression would be independent of Corto, since *corto* mutants never present truncated veins [15].

## Conclusion

Our results suggest that the Elongin complex might participate in determination of vein and intervein cell identity during wing development. We propose that this complex might interact with the ETP Corto at certain target genes and fine-tune their transcription in a cell-type specific manner. One of these targets could be the vein-promoting gene *rho*. In intervein cells, binding of Corto to the Elongin complex could prevent transcription of *rho*. Corto could also recruit other chromatin factors, such as the BAP chromatin-remodeling complex that was previously shown to inhibit *rho* expression in intervein cells. By contrast, in vein cells, the Elongin complex could participate in *rho* transcriptional elongation independently of Corto.
